# Performance evaluation of frequency division duplex (FDD) massive multiple input multiple output (MIMO) under different correlation models

**DOI:** 10.7717/peerj-cs.1017

**Published:** 2022-06-21

**Authors:** Alaa M. Abdul-Hadi, Marwah Abdulrazzaq Naser, Muntadher Alsabah, Sadiq H. Abdulhussain, Basheera M. Mahmmod

**Affiliations:** 1 Department of Computer Engineering, University of Baghdad, Al-Jadriya, Baghdad, Iraq; 2 Department of Architectural Engineering, University of Baghdad, Al-Jadriya, Baghdad, Iraq; 3 Department of Electronic and Electrical Engineering, University of Sheffield, Sheffield, United Kingdom

**Keywords:** Massive-MIMO, Frequency-division-duplex, Correlation model

## Abstract

Massive multiple-input multiple-output (massive-MIMO) is considered as the key technology to meet the huge demands of data rates in the future wireless communications networks. However, for massive-MIMO systems to realize their maximum potential gain, sufficiently accurate downlink (DL) channel state information (CSI) with low overhead to meet the short coherence time (CT) is required. Therefore, this article aims to overcome the technical challenge of DL CSI estimation in a frequency-division-duplex (FDD) massive-MIMO with short CT considering five different physical correlation models. To this end, the statistical structure of the massive-MIMO channel, which is captured by the physical correlation is exploited to find sufficiently accurate DL CSI estimation. Specifically, to reduce the DL CSI estimation overhead, the training sequence is designed based on the eigenvectors of the transmit correlation matrix. To this end, the achievable sum rate (ASR) maximization and the mean square error (MSE) of CSI estimation with short CT are investigated using the proposed training sequence design. Furthermore, this article examines the effect of channel hardening in an FDD massive-MIMO system. The results demonstrate that in high correlation scenarios, a large loss in channel hardening is obtained. The results reveal that increasing the correlation level reduces the MSE but does not increase the ASR. However, exploiting the spatial correction structure is still very essential for the FDD massive-MIMO systems under limited CT. This finding holds for all the physical correlation models considered.

## Introduction

Global wireless data-traffic has grown dramatically in the last years. According to recent statistics, monthly data-traffic would exceed 607 Exabytes (EBs) by 2025 (IMT, 2015). To this end, sixth-generation (6G) wireless communications networks are being developed to accommodate the substantial growth in mobile data-traffic (Chen et al., 2020; Chowdhury et al., 2020). Specifically, 6G wireless communication networks aim to increase the data rate, support 4K video streaming, exploit massive Machine-to-Machine (M2M) devices, increase link reliability, and reduce latency (Dang et al., 2020; Agiwal, Roy & Saxena, 2016; Aljiznawi et al., 2017). However, in order to meet these requirements, advanced physical layer technologies are still required. Massive multiple-input multiple-output(massive-MIMO) is to date considered as a promising technology to meet the huge requirements for high data rates in the future the 6G networks (Han, Jornet & Akyildiz, 2018; De Carvalho et al., 2020). Unlike traditional MIMO techniques, massive-MIMO systems allow the base station (BS) to deploy a large number of antennas in order to increase the data rate, enhance link reliability, and achieve energy efficiency (Rusek et al., 2013; Ngo, Larsson & Marzetta, 2013; Malkowsky et al., 2017; Han, Jornet & Akyildiz, 2018; De Carvalho et al., 2020). Furthermore, massive-MIMO systems have the potential to improve the array gain and link budget. To this end, sufficiently accurate channel state information (CSI) for downlink (DL) precoding is need to achieve the full potential gain of massive-MIMO systems. However, deploying large arrays at the BS incurs an unacceptably large training overhead especially when the coherence time (CT) is short. Therefore, finding a feasible solution for achieving sufficiently accurate CSI estimation under short CT is essential. The majority of massive-MIMO works have forced on time division duplex (TDD) transmission due to the technical challenge of CSI estimation, see *e.g.*, Jose et al. (2011), Ngo, Larsson & Marzetta (2013), Hoydis, Ten Brink & Debbah (2013), Yang & Marzetta (2013), Zuo et al. (2016), Li et al. (2019). The uplink (UL) and DL channels are considered to be reciprocal in TDD operation mode, hence allowing the UL CSI estimation to be exploited for DL precoding without requirements for the DL CSI estimation. However, the calibration error and hardware impairments are to date considered as severe limitations for the TDD systems in practice (Kaltenberger et al., 2010; Bjrnson et al., 2013; Mi et al., 2017; Vieira et al., 2017). Furthermore, most of current mobile systems operate in the frequency division duplex (FDD) mode. Thus, there is a crucial requirement for enabling FDD operation mode. However, the DL and UL channels in FDD systems are non-reciprocal, and hence, CSI estimation using UL training sequence can not be achieved in FDD systems (Tse & Viswanath, 2005). Specifically, to achieve a minimum mean-square-error (MSE) in the FDD systems, users must estimate the DL CSI of each of the *N* BS antennas (Hassibi & Hochwald, 2003; Rusek et al., 2013; Björnson, Larsson & Marzetta, 2016). In massive-MIMO systems with enormous antenna arrays at the BS, the training overhead becomes excessive. As such, CSI estimation becomes extremely difficult, especially when CT is short (Björnson, Larsson & Marzetta, 2016; Alsabah, Vehkapera & O’Farrell, 2020; Naser et al., 2020). This is because the DL training sequence for CSI estimation would take up most of the CT allocated period, leaving no time for delivering useful data to the users. Therefore, finding a practical CSI estimate design with a shorter training sequence length is critical for FDD systems with short CT.

Several works have been proposed to address the challenge of CSI estimation overhead in FDD massive-MIMO systems, see *e.g.*, Adhikary et al. (2013), Nam, Caire & Ha (2017), Rao & Lau (2014), Gao et al. (2015), Gao et al. (2016), Han, Lee & Love (2017), Choi, Love & Bidigare (2014), Noh et al. (2014), So et al. (2015). In particular, the works in Adhikary et al. (2013) and Nam, Caire & Ha (2017) develop a two-stage precoding method for DL CSI estimation, termed as joint-spatial-division-and-multiplexing (JSDM). In these works, the users can be divided into groups where the BS can select a specific group to be served. Then, a spatial multiplexing technique can be used to server the selected groups. The authors found that the pilot length for CSI estimation can be increased proportionally with the number of groups and not with *N*. As such, the overhead in FDD systems can be reduced. While the works in Adhikary et al. (2013) and Nam, Caire & Ha (2017) try to reduce the overhead of CSI estimation, advanced scheduling algorithms become essential for grouping the users. Furthermore, the works in Adhikary et al. (2013) and Nam, Caire & Ha (2017) are not considered the challenge of DL training with single-stage precoding. As such, these works are not able to predict the FDD massive-MIMO performance when a short CT is considered. The works in Rao & Lau (2014), Gao et al. (2015), Gao et al. (2016) and Han, Lee & Love (2017) consider the compressed sensing (CS) based techniques to reduce the CSI estimation overhead in FDD systems; while the works in Choi, Love & Bidigare (2014) and Noh et al. (2014); So et al. (2015) exploit the temporal correlation based on Kalman filter and spatial correlation to reduce the CSI estimation overhead in FDD systems. Although, the aforementioned works try to address the challenge of training sequence design for CSI estimation, the algorithms used are still computationally complex. In addition, future wireless communications networks aim to be operated in scenarios with high frequencies and mobilities, where both required a short CT. As such, a sufficiently accurate CSI estimation for FDD massive-MIMO systems with limited CT is essential. The authors in Wagner et al. (2012), Zheng et al. (2019) and Mostafa, Newagy & Hafez (2021) investigate UL CSI estimation with TDD systems using ZF precoding. They found that the number of UL sequences is proportional to the number of users and independent of the number of BS antennas *N* that can be made as large as required. However, there is a need to investigate the ZF precoding in FDD systems. The authors in Amadid et al. (2022) and Boulouird et al. (2022) consider the spatially correlated channel in TDD protocol. Therefore, there is an essential requirement to investigate the FDD massive-MIMO systems with limited CT considering different spatial correlation models. Furthermore, the achievable sum rate (ASR) performance in the typical wireless networks is greatly influenced by the received signal and by the level of correlation. While much focus is dedicated to examine the received signal effect on ASR, little consideration is given to examine the correlation effect on ASR. Furthermore, the uncorrelated channel cases have been investigated in the majority of massive-MIMO studies, see *e.g.*, Jose et al. (2011), Marzetta et al. (2016), Chen, Guevara & Pollin (2017), Liu et al. (2017), Ngo, Larsson & Marzetta (2013). A common conclusion arising from the aforementioned works is that a necessary condition for channel hardening can be achieved with massive-MIMO over which the variance goes to zero. However, this is not the case when the channel is highly correlated. Spatial channel correlation model is essential for evaluating the performance of any wireless communications systems. This is because it can reflect the propagation characteristics of the signals in practical radio environments. Therefore, this article aims to investigate the massive-MIMO performance using different channel correlation models namely the *P*-DoF, the Weichselberger model (Weichselberger et al., 2006), the one ring (OR) developed by Jakes in Jakes & Cox (1994), Laplacian (Lap) (Molisch, 2012), and exponential  (Loyka, 2001; Albdran et al., 2016; Albdran, Alshammari & Matin, 2017; Croisfelt Rodrigues, Marinello & Abrão, 2019) channel models.

### Research contributions and findings:

In this article, the performance of FDD massive-MIMO systems using five different channel correlation models is investigated. To this end, the physical structure of the spatial correlation matrix is exploited to design an efficient sequence for CSI estimation in DL FDD systems. A realistic scenario, where the number of BS antennas is larger than the CT, is considered. Using the proposed training design, the overhead in FDD massive-MIMO systems can be significantly decreased by utilizing the correlation structure in the training sequence design. This article includes the following contributions as a result of this research:

• The majority of MIMO research has considered that the channels are simulated with uncorrelated fading, which appears to be a rather unrealistic consideration. Therefore, the goal of this research is to investigate the performance of massive-MIMO systems in a practical situation with spatially correlated channels and a short CT. Specifically, this article investigates a practical massive-MIMO setting considering five different spatial correlation models and assumes a limited CT.
• This article addresses the challenge of designing feasible training sequence for sufficiently accurate DL CSI estimation in FDD systems with limited CT. Therefore, a low-complexity subspace MMSE CSI estimation, which utilizes the effective eigenvectors of correlation matrix with the dominated eigenvalues, is considered. To this end, the low dimensions subspace is exploited to reduce the DL CSI estimation overhead in FDD systems.
• This article seeks to explain how many BS antennas are required to approach channel hardening under different correlation models.
• Unlike previous research works that have investigated the system performance using the MSE only, this article investigates the FDD massive-MIMO systems considering both the MSE and ASR with short CT, which are essential metrics for many wireless systems applications.
• This article considers a zero-forcing (ZF) precoding technique because it has the ability to suppress the interference with high SNR values in comparison to the conventional precoding scheme such as matched filter.
• This article also develops an analytical solution for the MSE, which is valid for all the correlation models.
• Comparisons between the ASR using the proposed DL CSI estimation design and the perfect CSI estimation are carried our based on *P*-DoF, Weichselberger, one ring (OR), laplacian, and the exponential channel models.

The results show that increasing spatial correlation is favorable to CSI estimation, as evidenced by the MSE being minimized. This is attributed to the fact that increasing the level correlation reduces channels dimensions, and hence reducing the MSE. Furthermore, when the correlation level is increased, the power is concentrated in a few directions. Hence, there is a need for low dimensions channel estimation with reduced CSI overhead. The results also show that with perfect CSI estimation when the spatial correlation increases, the ASR is degraded. However, with imperfect CSI estimation considering short CT, increasing the spatial correlation is very beneficial to the ASR. Furthermore, the results show that, unlike the uncorrelated fading channel, in high correlation channel, it is hard to achieve channel hardening with zero variance even with large *N*. Specifically, the results demonstrate that with a strong spatial correlation, a large loss in channel hardening can be obtained. This article also presents some important insights about the FDD massive-MIMO systems with various correlation models. Finally, this article makes several suggestions for future research that could lead to new research possibilities.

*Article organization*: ‘System Model’ introduces the system model. Then, ‘Downlink Csi Estimation and Training Sequence Design’ discusses the DL CSI estimation and training sequence design. ‘Data Transmission with Linear Precoder’ introduces the ASR and precoding design. After that, different physical correlation models are described in ‘Physical Spatial Correlation Models’. In ‘Channel Hardening in Massive-Mimo Systems’ the channel hardening effect is discussed. The experiment results are then presented in ‘Numerical Results and Performance Evaluation’. Finally, this article is concluded in ‘Concluding Remarks and Future Research Directions’.

*Notations:* This article uses bold upper case for matrix and a bold lower case character for a vector. We use ||**A**||_F_ for the Frobenius-norm operation and tr(**A**) for the trace. This article also considers **A**^T^, **A**^H^, and (**A**)^−1^ for the operation of transpose, Hermitian, and matrix inversion **A**, respectively. The (**A**⊗**B**) denotes the Kronecker product of **A** and **B** and $\mathcal{CN}(\mathrm{&mu;},\mathbf{R})$ represents the Gaussian-distribution where µstands for the mean and **R** for the covariance matrix at the BS.

## System Model

**Figure 1:**
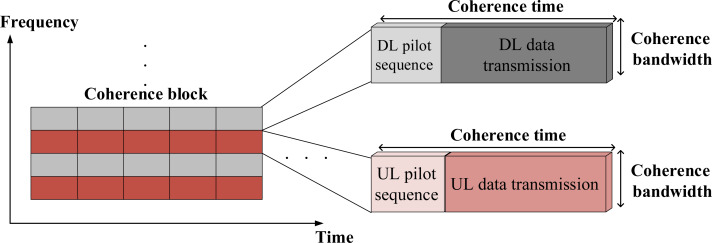
The illustration of the resource block in time and frequency.

**Figure 2:**
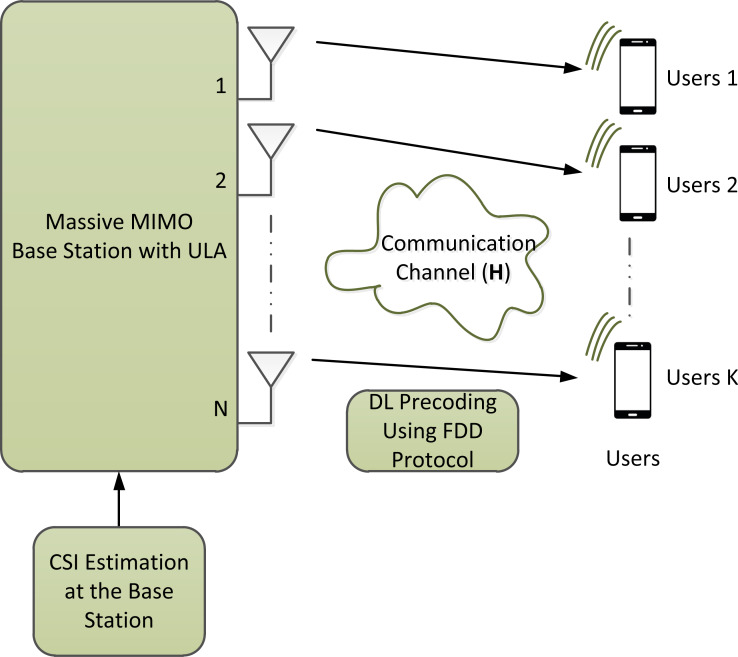
The illustration of the system model considered in this article.

This present article assumes a single-cell wireless communication system. A Rayleigh fading channel is considered in this article. However, finding an efficient training sequence design in Rician fading channel (Amadid, Belhabib & Zeroual, 2022) can be considered in the future. We would like to emphasis that our article discusses a uniform linear array and not three-dimensional array. However, three-dimensional array, *e.g.*, Amadid, Boulouird & Riadi (2022), can be considered in the future. A time-varying channel is considered, which is partitioned into several coherence blocks. To this end, a block-fading model with time and frequency resources is used, over which the channels are assumed to be frequency flat (Alsabah, 2020). The time slot, however, which is enumerated in symbols per transmission block, corresponds to channel CT *τ*_c_. The channel CT is partitioned into the training phase to enable DL CSI estimation, and the data phase for useful data transmission as shown in Fig. 1. The transmit power is equally distributed between the training and data phases. We assume massive-MIMO system with a base station (BS) that uses *N* antennas. The BS is communicated with *K* users over the same time and frequency resources assuming *N* ≫ *K*. Furthermore, this article considers a single-stage precoding. Fig. 2 shows the system model considered in this article with FDD protocol.

## Downlink CSI estimation and training sequence design

In the typical FDD systems, the BS needs to perform a precoding based on DL CSI estimation to serve multiple users, simultaneously. During the CSI estimation phase, the BS transmits DL sequences of length *τ*_p_ with training power denoted by *ρ*_p_. The rest of the time is spent on data transmission, which is given as *τ*_d_ = *τ*_c_ − *τ*_p_. This article focuses on minimizing the training sequence length *τ*_p_ over a limited CT *τ*_c_. To this end, this article constructs the training sequences in the DL based on the dominant eigenvectors of the transmit correlation matrix. To obtain the CSI estimation of the DL channel, the BS sends training sequences of length *τ*_p_. The training sequence matrix is denoted by **S**_p_ ∈ ℂ^*N*×*τ*_p_^. This training sequence must meet the energy constraint. This implies that $\mathrm{tr} \left( \right. {\mathbf{S}}_{\mathrm{p}}^{\text{H}}{\mathbf{S}}_{\mathrm{p}} \left( \right. ={\rho }_{\mathrm{p}}{\tau }_{\mathrm{p}}$. This article considers a scenario where the users have common spatial correlation matrices. In addition, this article assumes an equal power allocation between the training and data phases, *i.e., ρ*_p_ = *ρ*_d_. However, non-uniform power allocations across the users and optimization with respect to different chooses of *ρ*_p_ and *ρ*_d_ could be considered in the future. Therefore, the *k*-th user received training signal, **y**_*k*_ ∈ ℂ^*τ*_p_^, can be expressed as

(1) \begin{eqnarray*}{\mathbf{y}}_{k}={\mathbf{S}}_{\mathrm{p}}^{\mathrm{H}}{\mathbf{h}}_{k}+{\mathbf{n}}_{k},\end{eqnarray*}

where **n**_*k*_ ∈ ℂ^*τ*_p_^ represents the receiver noise. The received noise is assumed to be complex Gaussian with $\mathcal{CN}$$ \left( \right. {\mathbf{0}}_{\mathrm{}},{\mathbf{I}}_{{\tau }_{\mathrm{p}}} \left( \right. $ and **h**_*k*_ ∈ ℂ^*N*^ ∼ $\mathcal{CN}$$(\mathbf{0},{\mathbf{R}}_{\mathrm{k}}^{\mathrm{}})$ denotes the complex baseband DL channel vector, which can be represented using Karhunen–Loeve method as

(2) \begin{eqnarray*}{\mathbf{h}}_{k}={\mathbf{R}}_{k}^{1/2}{\tilde {\mathbf{h}}}_{k},\end{eqnarray*}

where ${\tilde {\mathbf{h}}}_{k}\sim $$\mathcal{CN}$(**0**, **I**_N_) denotes the DL channel and satisfies ${\mathbf{R}}_{k}=E[{\mathbf{h}}_{k}{\mathbf{h}}_{k}^{\text{H}}]\in {\mathbb{C}}^{N\times N}$. The spatial correlation matrix **R**_*k*_ has an eigenvalue-decomposition of

(3) \begin{eqnarray*}{\mathbf{R}}_{k}={\mathbf{T}}_{k}{\Delta }_{k}{\mathbf{T}}_{k}^{\mathrm{H}},\end{eqnarray*}

where **T**_*k*_ = [**t**_*k*,1_, …, **t**_*k*,*N*_] ∈ ℂ^*N*×*N*^ represents the eigenvectors of **R**_*k*_ and **Δ**_*k*_ denotes the eigenvalues of **R**_*k*_, which are arranged as *δ*_*k*,1_ ≥ *δ*_*k*,2_ ≥ ⋯ ≥ *δ*_*k*,*N*_. The correlation matrix is considered as a large-scale channel statistics, which are assumed to be frequency invariant, and hence, can be efficiently obtained at both transceivers sides (Xie et al., June-2018). Since the channel statistics are assumed to be known, linear filter that exploits the channel statistics is considered. Hence, an optimized CSI estimation in the DL with *τ*_p_ < *N* can be obtained using Bayesian estimation. An example of Bayesian estimation is the minimum mean square error (MMSE) CSI estimation **G**_*k*_. The MMSE filter uses the statistics of channel and the statistics of noise (Kay, 1993).

(4) \begin{eqnarray*}{\mathbf{G}}_{k}={\mathbf{R}}_{k}{\mathbf{S}}_{\mathrm{p}} \left( \right. {\mathbf{S}}_{\mathrm{p}}^{\mathrm{H}}{\mathbf{R}}_{k}{\mathbf{S}}_{\mathrm{p}}+{\mathbf{I}}_{{\tau }_{\mathrm{p}}}{ \left( \right. }^{-1}.\end{eqnarray*}

Accordingly, the resulting channel estimate ${\hat {\mathbf{h}}}_{k}\sim $ $\mathcal{CN}(\mathbf{0},{\mathrm{&Theta;}}_{k})$ is obtained as

(5) \begin{eqnarray*}{\hat {\mathbf{h}}}_{k}={\mathbf{G}}_{k}{\mathbf{y}}_{k}.\end{eqnarray*}

where **y**_*k*_ is the received training signal in Eq. (1). In what follows, the MSE of the channel estimation that can be calculated using Monte Carlo simulation is provided as

(6) \begin{eqnarray*}{\text{MSE}}_{\text{sim}}=\sum _{k=1}^{K}E{|}{|}{\mathbf{h}}_{k}-{\hat {\mathbf{h}}}_{k}{|}{{|}\nolimits }^{2},\end{eqnarray*}

where (sim) refers to Monte Carlo simulation that is used to calculate the simulated MSE. The expression of the MSE in Eq. (6) is valid due to the orthogonality principle between the channel estimation error ${\hat {\mathbf{h}}}_{k}$ and the channel estimation ${\tilde {\mathbf{h}}}_{k}$, where both channels are typically considered to be uncorrelated. Accordingly, the per user channel estimation error vector, which is independent of ${\hat {\mathbf{h}}}_{k}$, can be expressed as

(7) \begin{eqnarray*}{\mathbf{e}}_{k}={\mathbf{h}}_{k}-{\hat {\mathbf{h}}}_{k}.\end{eqnarray*}

Let define the per user error covariance matrix as **C**_*e*,*k*_ ∈ ℂ^*N*×*N*^, which can be expressed as

(8) \begin{eqnarray*}{\mathbf{C}}_{e,k}=\mathbb{E}\, \left\{ \right. \,{\mathbf{e}}_{k}{\mathbf{e}}_{k}^{\mathrm{H}} \left( \right. ,\end{eqnarray*}

(9) \begin{eqnarray*}=\mathbb{E} \left\{ \right. \left( \right. {\mathbf{h}}_{k}-{\hat {\mathbf{h}}}_{k} \left( \right. \left( \right. {\mathbf{h}}_{k}-{\hat {\mathbf{h}}}_{k}{ \left( \right. }^{\mathrm{H}} \left( \right. ,& \end{eqnarray*}

(10) \begin{eqnarray*}= \left( \right. {\mathbf{R}}_{k}-{\mathbf{R}}_{k}{\mathbf{S}}_{\mathrm{p}} \left( \right. {\mathbf{S}}_{\mathrm{p}}^{\mathrm{H}}{\mathbf{R}}_{k}{\mathbf{S}}_{\mathrm{p}}+{\mathbf{I}}_{{\tau }_{\mathrm{p}}}{ \left( \right. }^{-1}{\mathbf{S}}_{\mathrm{ p}}^{\mathrm{H}}{\mathbf{R}}_{k} \left( \right. .& \end{eqnarray*}

The MMSE of the CSI estimation should satisfy $\Theta =\mathbb{E} \left\{ \right. {\hat {\mathbf{h}}}_{k}{\hat {\mathbf{h}}}_{k}^{\mathrm{H}} \left( \right. $. As such, the MMSE of the CSI estimation can be expressed as

(11) \begin{eqnarray*}{\mbrm{\Theta }}_{k}={\mathbf{R}}_{k}{\mathbf{S}}_{\mathrm{p}} \left( \right. {\mathbf{S}}_{\mathrm{p}}^{\mathrm{H}}{\mathbf{R}}_{k}{\mathbf{S}}_{\mathrm{p}}+{\mathbf{I}}_{{\tau }_{\mathrm{p}}}{ \left( \right. }^{-1}{\mathbf{S}}_{\mathrm{ p}}^{\mathrm{H}}{\mathbf{R}}_{k}.\end{eqnarray*}

The expression in Eq. (10) is minimized by maximizing Eq. (11), which represents the MMSE of the CSI channel estimation. To this end, the MSE can be analytically expressed as

(12) \begin{eqnarray*}{\text{MSE}}_{\text{an}}=\sum _{k=1}^{K}\mathrm{tr}({\mathbf{C}}_{e,k}),\end{eqnarray*}

where tr stands for the trace of a matrix, which represents the sum of its eigenvalues. The subscript (an) stands for the analytical form of the MSE. The expression in Eq. (12) provides the output of the CSI estimator, which minimizes the MSE. The expression in Eq. (12) implies that the overall MSE performance relies on the eigenvalues of the expression in Eq. (10), which characterizes the overall MSE. Using the well known matrix inversion Lemma, the formulation of the error covariance matrix in Eq. (10) can be simplified to

(13) \begin{eqnarray*}{\mathbf{C}}_{e,k}= \left( \right. {\mathbf{R}}_{k}^{-1}+{\mathbf{S}}_{\mathrm{ p}}{\mathbf{S}}_{\mathrm{p}}^{\mathrm{H}}{ \left( \right. }^{-1},\end{eqnarray*}

where **S**_p_ should satisfy the energy constraint, which is given as $\text{tr}({\mathbf{S}}_{\mathrm{p}}^{\mathrm{H}}{\mathbf{S}}_{\mathrm{p}})={\tau }_{\mathrm{p}}{\rho }_{\mathrm{p}}$. Clearly, the expression in Eq. (13) relies on the correlation matrix **R**_*k*_, the sequence matrix **S**_p_, the training sequence length *τ*_p_, and the training power *ρ*_p_. The expression in Eq. (13) motivates the use of the structure of the correlation matrix in the DL training design.

### Training sequence design

In FDD systems, the BS sends the DL pilot sequence to the users. Then, the users quantize the received signal and send it back to the BS to estimate the DL channels. The BS is then performed a DL precoding based on the CSI estimation. In other instances, each user has the ability to estimate their DL channel. As such, the user returns the quantized CSI estimation to the BS. In what follows, we explain the training sequence design, which is required to achieve the highest ASR.

The massive-MIMO channels could be highly correlated. Due to this correlation most of the eigenvalues become closed to zero. Therefore, the channels can be divided into a few number of dimensions (the eigenvalues that are not closed to zero), which can be smaller than *N*. Besides, a significant percentage of the energy would be focused in a few strong spatial directions rather than dispersed evenly. Therefore, more power should be assigned to those spatial directions and zero power can be allocated to the weakest directions. This observation motivated the authors to explore the structure of the correlation matrix **R**_*k*_ in the pilot design to reduce the overhead of DL CSI estimation in the FDD systems. This article proposes a computationally efficient pilot design.

Following the approach of majorization theory, the MSE of CSI estimation can be decreased considerably by the increasing spatial correlations. Therefore, in the presence of correlation, the sequence length can be chosen to be less than *N*. This is especially crucial for the FDD massive-MIMO systems, where the MSE must be kept to a minimum and the ASR must be maximized with the shortest sequence length possible. In this article, the training matrix **S** is designed based on the correlation matrix. Specifically, the dominated eigenvectors the correlation matrix is used in the DL sequence construction in order to reduce the DL CSI estimation overhead. We aim to maximize the DL ASR of an FDD massive-MIMO system.

Let consider a scenario where each user has the same statistical denoted by the correlation matrix, *i.e.,* **R**_*k*_ = **R**. Based on this consideration, **R** can be decomposed as

(14) \begin{eqnarray*}\mathbf{R}=\mathbf{T}\Delta {\mathbf{T}}^{\mathrm{H}},\end{eqnarray*}

where **T** = [**t**_1_, …, **t**_*N*_] ∈ ℂ^*N*×*N*^ denotes a unitary matrix that contains the eigenvectors of the correlation matrix **R** and Δ represents the eigenvalues of the the correlation matrix **R** that are arranged as *δ*_1_ ≥ *δ*_2_ ≥ ⋯ ≥ *δ*_*N*_. In this article, the dominate singular vectors from left unitary matrix is extracted to reduce the CSI estimation overhead. This sequence is designed to build the subspace projection matrix that achieves a minimum MSE with less training sequence length. This implies that with the proposed training sequence design, the DL channel will be estimated in the reduced dimension subspace. Specifically, training matrix **S**_p_ ∈ ℂ^*N*×*τ*_p_^ is designed using the dominated eigenvectors of **R** as given in Eq. (15).

(15) \begin{eqnarray*}{\mathbf{S}}_{\mathrm{p}}= \left[ \right. {\mathbf{t}}_{1},\ldots ,{\mathbf{t}}_{{\tau }_{\mathrm{p}}} \left( \right. \end{eqnarray*}

As such, the training matrix **S**_p_ should satisfy ${\mathbf{S}}_{\mathrm{p}}^{\mathrm{H}}{\mathbf{S}}_{\mathrm{p}}={\mathbf{I}}_{{\tau }_{\mathrm{p}}}$. To reduce the training overhead, the eigenvectors **t**_*τ*_p_+1_, …, **t**_*N*_ of **R**, is not used in the CSI estimation. Proper power loading across the beams directions **Δ** can be considered in the future. We start by substituting the training sequence design in Eq. (15) into Eq. (11). Hence, by exploiting the eigenvalue decomposition method, the MMSE CSI estimate can be simplified with some straightforward algebra as

(16) \begin{eqnarray*}\Phi ={\rho }_{\mathrm{p}}{\mathbf{T}}_{{\tau }_{\mathrm{p}}}\Delta 2\tau \mathrm{p} \left( \right. {\rho }_{\mathrm{p}}\Delta \tau \mathrm{p}+{\mathbf{I}}_{{\tau }_{\mathrm{p}}}{ \left( \right. }^{-1}{\mathbf{T}}_{{\tau }_{\mathrm{p}}}^{\mathrm{H}},\end{eqnarray*}

where **Δ***τ*_p_ ∈ ℝ^*τ*_p_×*τ*_p_^ is a diagonal matrix with eigenvalues that are arranged in a descending way as *δ*_1_ ≥ *δ*_2_ ≥ ⋯ ≥ *δ*_*τ*_p__. In what follows, an analytical solution for the MSE of CSI estimation in the FDD systems is given. Let substitute the expression in Eq. (16), which stands for the MMSE of CSI estimation, into Eq. (12). To this end, an analytical mean square error MSE_an_ can be written as

(17) \begin{eqnarray*}{\text{MSE}}_{\mathrm{an}}=\text{tr} \left( \right. \mathbf{R}-{\rho }_{\mathrm{p}}{\mathbf{T}}_{{\tau }_{\mathrm{p}}}{\Delta }_{\tau \mathrm{p}}^{2} \left( \right. {\rho }_{\mathrm{ p}}{\Delta }_{{\tau }_{\mathrm{p}}}+{\mathbf{I}}_{{\tau }_{\mathrm{p}}}{ \left( \right. }^{-1}{\mathbf{T}}_{{\tau }_{\mathrm{p}}}^{\mathrm{H}} \left( \right. .\end{eqnarray*}

By exploiting the eigenvalue decomposition **T** in Eq. (14), with straightforward algebra and matrix analysis in Petersen & Michael (2008) , the formulation of the MSE CSI estimation can be simplified to

(18) \begin{eqnarray*}{\text{MSE}}_{\text{an}}=\text{tr} \left( \right. {\mathbf{T}}_{{\tau }_{\mathrm{p}}} \left( {\Delta }_{{\tau }_{\mathrm{p}}}-{\rho }_{\mathrm{p}}{\Delta }_{{\tau }_{\mathrm{p}}}({\rho }_{\mathrm{p}}\Delta +{\mathbf{I}}_{{\tau }_{\mathrm{p}}})^{-1}{\Delta }_{{\tau }_{\mathrm{p}}} \right) {\mathbf{T}}_{{\tau }_{\mathrm{p}}}^{\mathrm{H}} \left( \right. .\end{eqnarray*}

By exploiting the trace operation, the formulation in Eq. (18) can be expressed as

(19) \begin{eqnarray*}{\text{MSE}}_{\text{an}}=\text{tr} \left( \right. {\Delta }_{{\tau }_{\mathrm{p}}}-{\rho }_{\mathrm{p}}{\Delta }_{{\tau }_{\mathrm{p}}}({\rho }_{\mathrm{p}}\Delta +{\mathbf{I}}_{{\tau }_{\mathrm{p}}})^{-1}{\Delta }_{{\tau }_{\mathrm{p}}} \left( \right. .\end{eqnarray*}

Exploiting some algebra manipulation allows the formulation of the MSE of CSI estimation in Eq. (19) to be simplified to

(20) \begin{eqnarray*}{\text{MSE}}_{\text{an}}=\sum _{m=1}^{{\tau }_{\mathrm{p}}}{\delta }_{m}- \frac{{\rho }_{\mathrm{p}}\,{\delta }_{m}^{2}}{{\rho }_{\mathrm{p}}{\delta }_{m}+1} .\end{eqnarray*}

Thus, an analytical solution of the MSE based on the CSI estimation can be expressed in a simplified form as

(21) \begin{eqnarray*}{\text{MSE}}_{\text{an}}=\sum _{m=1}^{{\tau }_{\mathrm{p}}} \frac{{\delta }_{m}}{{\rho }_{\mathrm{p}}{\delta }_{m}+1} .\end{eqnarray*}

The formulation in Eq. (21) holds for any correlation models. According to the expression in Eq. (21), increasing the training power would reduce the estimation error. Another finding from Eq. (21) is that, increasing the correlation would also reduce the estimate error. The data transmission utilizes linear precoding at the transmitter. The received signal is discussed in the next section.

We would like to emphasis that the accuracy of channel estimation is improved by minimizing the estimation error, which is denoted by the MSE performance metric. In order to understand the fundamental impact of spatial correlation on the MSE of channel estimate, the basic concept of majorization theory is presented here.

Majorization (Marshall, Olkin & Arnold, 1979): Let **x** = [**x**_1_, …, **x**_*N*_]^T^ and **z** = [**z**_1_, …, **z**_*N*_]^T^ be as two positive real-valued vectors, containing the elements of **x** and **z**, respectively, which are arranged in descending order. Vector **x** majorizes **z** (**x**⪰**z**) if

(22) \begin{eqnarray*}\sum _{i=1}^{n}\mathbf{x}\geq \sum _{i=1}^{n}\mathbf{z},\end{eqnarray*}

for all 1 ≤ *n* < *N*. If vectors **x** and **z** contain eigenvalues of the channel covariance matrices, then by the majorization property, **z** is less spread out than **x** and vector **x** is more spatially correlated than vector **z**. Similarly, for the MSE cost function under consideration, *C*_*e*_(**x**) ≤ *C*_*e*_(**z**), which implies that the MSE of the stronger eigenvalues are less than the MSE of the weakest eigenvalues. As such, high spatial correlation reduces the estimation error and the eigendirections of the channel covariance matrix, with large eigenvalues have a smaller estimation error variance than the eigendirections with smaller eigenvalues. Specifically, based on the majorization theory, the MSE performance decreases with increasing spatial channel correlations. In addition to the principle discussed above, the expression (21) indicates that increasing the correlation would reduce the estimate error, *i.e.,* MSE. The results presented in our article also confirm that the MSE of CSI estimation is reduced when the spatial correlation is increased.

## Data transmission with linear precoder

This section presents the data transmission using linear precoding technique. To this end, the *k*-th user received data signal can be expressed as

(23) \begin{eqnarray*}{y}_{k}=\sqrt{{\rho }_{\mathrm{d}}}\,{\mathbf{h}}_{k}^{\mathrm{T}}\mathbf{V }\mathbf{s}+{n}_{k},\end{eqnarray*}

where the vector **h**_*k*_ ∈ ℂ^*N*^ is the DL instantaneous channel and **s** = [*s*_1_, …, *s*_*K*_]^T^ ∈ ℂ^*K*^ denotes the vector of data symbols, which is considered to be an independently and identically distributed and satisfies 𝔼[**ss**^H^] = **I**_*K*_, and **V** = [**v**_1_, …, **v**_*K*_] ∈ ℂ^*N*×*K*^ is the precoding matrix at the BS. Parameter *n*_*k*_ denotes the received noise, which is also considered to be an independently and identically distributed. Therefore, the received SINR, denoted as γ_*k*_, can be expressed as

(24) \begin{eqnarray*}{\mathrm{\gamma }}_{k}= \frac{\mid {\mathbf{h}}_{k}^{\mathrm{T}}{\mathbf{v}}_{k}{\mid }^{2}}{ \frac{1}{{\rho }_{\mathrm{d}}} +\sum _{l\not = k}^{K}{|}\,{\mathbf{h}}_{k}^{\mathrm{T}}{\mathbf{v}}_{l}\,{{|}}^{2}} .\end{eqnarray*}

where *ρ*_d_ denotes the SNR, which denotes the transmit power in the data transmission. To this end, zero forcing (ZF) precoder is considered, and hence, the normalized ZF precoding is given as

(25) \begin{eqnarray*}{\mathbf{v}}_{k}=\sqrt{ \frac{{p}_{k}}{ \left\vert \right. \left\vert \right. \left( \right. {\hat {\mathbf{H}}}^{\dagger }{ \left( \right. }^{:,k} \left\vert \right. { \left\vert \right. }_{2}^{2}} } \left( \right. {\hat {\mathbf{H}}}^{\dagger }{ \left( \right. }^{:,k},\end{eqnarray*}

where ${p}_{k}={|}{|}{\mathbf{v}}_{k}{|}{\mathop{{|}}\nolimits }_{2}^{2}$ is the power allocated to user *k*, which satisfies the transmit power constrain ${\mathop{\sum }\nolimits }_{k=1}^{K}{p}_{k}=1$. This normalization is used to guarantee that the per user transmit power to be constant in the data phase. The term ${\hat {\mathbf{H}}}^{\mathrm{T}}=[{\hat {\mathbf{h}}}_{1},\ldots ,{\hat {\mathbf{h}}}_{K}]$ considers the estimate of the downlink true channel, which is named in this article as (imperfect CSI), where (†) corresponds to the Moore–Penrose pseudo-inverse. With perfect channel estimation, which is named in this article as (perfect CSI), the precoder can be written as

(26) \begin{eqnarray*}{\mathbf{v}}_{\text{per},k}=\sqrt{ \frac{{p}_{k}}{ \left\vert \right. \left\vert \right. \left( \right. {\mathbf{H}}^{\dagger }{ \left( \right. }^{:,k} \left\vert \right. { \left\vert \right. }_{2}^{2}} } \left( \right. {\mathbf{H}}^{\dagger }{ \left( \right. }^{:,k},\end{eqnarray*}

where the notation (per) stands for perfect CSI estimation. As such, the SINR with perfect CSI can be written as

(27) \begin{eqnarray*}{\mathrm{\gamma }}_{\text{per},k}= \frac{\mid {\mathbf{h}}_{k}^{\mathrm{T}}{\mathbf{v}}_{\text{per},k}{\mid }^{2}}{ \frac{1}{{\rho }_{\mathrm{d}}} } .\end{eqnarray*}

Accordingly, the DL ASR with imperfect CSI estimation, denoted by Rate, is expressed as

(28) \begin{eqnarray*}\text{Rate}= \left( \right. \frac{{\tau }_{\mathrm{c}}-{\tau }_{\mathrm{p}}}{{\tau }_{\mathrm{c}}} \left( \right. \sum _{k=1}^{K}\mathbb{E} \left\{ \right. {\mathrm{log}}_{2} \left( \right. 1+{\mathrm{\gamma }}_{k} \left( \right. \left( \right. \text{[bit/s/Hz]}.\end{eqnarray*}

In addition, the DL ASR, with perfect CSI estimation, denoted by Rate_per_, is written as

(29) \begin{eqnarray*}{\text{Rate}}_{\text{per}}=\sum _{k=1}^{K}\mathbb{E} \left\{ \right. {\mathrm{log}}_{2} \left( \right. 1+{\mathrm{\gamma }}_{\text{per},k} \left( \right. \left( \right. \text{[bit/s/Hz]}.\end{eqnarray*}

The above expectation operation is obtained by taking into account all sources of randomness. A Monte Carlo simulation is used to determine the SINR. As previously stated, this work considers a practical correlation models. To this goal, the user channel’s elements are correlated rather than isotropically dispersed with Rayleigh fading model. This indicates that **h**_*k*_ has a strong spatial directionality. The received signal in Eq. (24) is dependent on the BS’s correlation, CSI estimation, and linear precoding strategy. However, due to the estimation error as well as the pre-log term, the CSI estimate imperfection lowers the ASR. The ASR maximizing will be discussed more in the following section.

### Formulation of the ASR maximization problem

Maximizing the DL ASR in the FDD massive-MIMO systems equates to the following optimization problem

(30) \begin{eqnarray*}\mathrm{maximize}_{{\tau }_{\mathrm{p}}} \left( \right. \frac{{\tau }_{\mathrm{c}}-{\tau }_{\mathrm{p}}}{{\tau }_{\mathrm{c}}} \left( \right. \sum _{k=1}^{K}\mathbb{E} \left\{ \right. {\mathrm{log}}_{2} \left( \right. 1+{\gamma }_{k} \left( \right. \left( \right. \nonumber\\\displaystyle \text{subject to} 1\leq {\tau }_{\mathrm{p}}\leq \mathrm{min} \left( \right. N,{\tau }_{\mathrm{c}} \left( \right. \end{eqnarray*}

Future 6G networks aim to maximize the ASR to fulfill the demands for increased data-traffic (Chen et al., 2020; Alsabah et al., 2021). Maximizing the ASR, in particular, is regarded one of the most important performance factors for future 6G networks (Chen et al., 2020; Alsabah et al., 2021). Hence, the goal of this research is to optimize the DL ASR in order to meet the growing need for high data rates. The ASR formulation in Eq. (30) illustrates that the training sequence length is important in maximizing the ASR. The SINR has a logarithmic effect on the ASR, but the training sequence duration has a linear effect on the ASR.

Remark 1 *As previously stated, the great majority of past research on FDD massive-MIMO systems has focused on determining the training sequence design and length that minimizes the MSE for a given training power. As such, choosing τ*_p_ = *N is not problematic in the conventional MIMO systems (Hassibi & Hochwald, 2003) since N is small. However, in massive-MIMO systems, N can be very large, which makes the DL CSI estimation problematic in FDD systems. To maximize the DL ASR in a scenario where N is larger than CT τ*_c_ *,the training sequence length τ*_p_* should be kept small. Noting that increasing τ*_p_ *minimizes the MSE of the DL CSI estimation. However, in short CT scenarios, as considered in this article, increasing τ*_p_ *reduces the DL ASR since the time remaining for transferring data to users is reduced. This motivates the use of statistical structure of massive-MIMO channel, which is captured by the physical correlation, for efficient DL CSI estimation with short CT τ*_c_.

The following section explains some well-known correlation models. The performance of FDD massive-MIMO systems is evaluated using these physical correlation models.

## Physical spatial correlation models

For modelling channel variations in conventional wireless communications systems, a random distribution is used. The channel statistics, which correspond to the random variable distributions, are commonly assumed to be available throughout the network (Björnson et al., 2017). As a result, Knowing the channel statistics is sufficient to design efficient communications systems (Stein, 1987; Björnson et al., 2017).

The uncorrelated Rayleigh fading model is a typical approach for modeling the covariance matrix (Marzetta et al., 2016). In Rayleigh fading model, the channel coefficients are considered to be independently and uniformly distributed. Besides, the signal Rayleigh fading model can be propagated across a wide range of scattering objects. Furthermore, in the Rayleigh fading model, the energy should be evenly distributed in all directions (Tse & Viswanath, 2005). However, the requirement for channel to be uncorrelated appears to be quite stringent. The channel coefficients in massive-MIMO systems are correlated because to the usage of a large arrays at the BS, which are closely spaced. In practice, the arrays face non uniform emission patterns and varying polarization. This makes the massive-MIMO channels correlated (Yu et al., 2004; Wallace & Jensen, 2001; Shepard et al., 2012; Gao et al., 2012). Besides, practical experiments in outdoor and indoor scenarios demonstrate that the MIMO channels are correlated, see, *e.g.*, Yu et al. (2004), Wallace & Jensen (2001), Shepard et al. (2012), Gao et al. (2012). As a result, considering more realistic correlation models are necessary to give a a closed to practice performance indication of massive-MIMO systems. Because of the high size of the array in massive-MIMO systems, the antenna spacing at the BS should be reduced, resulting in significant correlations. Due to this correlation, most of the eigenvalues become closed to zero. In the high correlation scenario, the channel covariance matrix would exhibit a large eigenvalue spread. In this case a large portion of the energy in the channel can be concentrated into a few directions, rather than being spread isotropically in all directions. Furthermore, antenna correlations are advantageous when it comes to channel estimation because of the possibility of rejecting the contamination in the estimates. If the contamination is reduced, then the MSE denoted can be reduced. For further discussion about this issue please refer to Heath Jr & Lozano (2018). A measure of the spatial correlation of channel is the eigenvalue distribution or what known as the eigenstructure of the channel covariance matrix. This indicates which spatial directions are statistically more likely to contain strong signal components than others. Strong spatial correlation is characterized by large eigenvalue variations, where the channel is confined to a small eigensubspace. Unlike the uncorrelated channels, with strong spatial correlation, a few eigenvalues dominate and hence the uncertainty in the channels can be significantly reduced.

To this end, spatial correlation models that reflect the practical propagation signals in radio environments should be considered. This is very essential for evaluating the performances of wireless communication systems. Therefore, this article aims to investigate the FDD massive-MIMO systems using the state-of-the-art spatial correlation models.

To reduce the systems overhead, signals can be propagated in the dominated angular domain that contains a fewer dimensions in compared to the number of BS antennas *N*. In order to reflect the spatial correlation in the massive-MIMO channels, different correlation models will be discussed in the following subsections. These channel correlation models will also be evaluated later in ‘Numerical Results and Performance Evaluation’.

### *P*-DoF model

The degrees-of-freedom (DoF) provided by the channel determines the correlation level. It can thus be represented by the *P*-subspace, where *P* can be denoted as the number of angular directions. The angular domain is divided into *P* directions, hence allowing a *P*-DoF to be performed. To this end, the correlation matrix **R** in the *P*-DoF model can be expressed as Hoydis, Ten Brink & Debbah (2013)

(31) \begin{eqnarray*}\mathbf{R}=\sqrt{ \frac{1}{c} }\mathbf{U}{\mathbf{A}}^{\mathrm{H}},\end{eqnarray*}

where the DoF is given as *P*/*N* = *c* ∈ (0, 1]. Also, matrix **U** ∈ ℂ^*K*×*P*^ is considered to be an an independent and identically distributed (i.i.d) with   $\mathcal{CN}(0,1)$. Matrix **A** ∈ ℂ^*N*×*P*^ can be constructed from *P* ≤ *N* of an *N* × *N* unitary matrix and satisfies **A**^H^**A** = **I**_*P*_. In this correlation model, **R** has rank *P*. The correlation parameter *c* is used to control the DoF/correlation in the massive-MIMO channel (Ngo, 2011; Payami & Tufvesson, 2012; Ngo, Larsson & Marzetta, 2013; Hoydis, Ten Brink & Debbah, 2013). The covariance matrix in *P*-DoF model is of the form $\mathbf{R}=\mathbb{E}[{\mathbf{h}}_{k}{\mathbf{h}}_{k}^{\text{H}}]= \frac{1}{c} \mathbf{A}{\mathbf{A}}^{\mathrm{H}}$.

### Weichselberger model

Weichselberger model (Weichselberger et al., 2006) alleviates the deficiencies of the Kronecker model by considering the joint correlation structure of both communication ends. As such, the average coupling between the spatial subchannels is effectively modeled. It is worth noting that in massive-MIMO system there is no correlation at the user’s side where the all users have a single antenna and all users are separable. Therefore, the matrix at the user side is considered to be an identity matrix. The covariance matrix in the Weichselberger can be written as

(32) \begin{eqnarray*}\mathbf{R}=\mathbf{U} \left( \right. \tilde {\mathbf{g}}\odot \tilde {\mathbf{h}} \left( \right. ,\end{eqnarray*}

where ⊙ denotes the element-wise (Hadamard) multiplication between two vectors and **h** is random channel with an independent and identically distributed (i.i.d.) distributed scaled by the coupling matrix $\tilde {\mathbf{g}}$ with *α*^*j*−1^, for different *α* that controls the degrees of correlation. Decreasing *α* means increasing spatial correlation. The coupling matrix has a full-rank and consists of real-valued non-negative elements. The coefficients of the coupling matrix specify the mean amount of energy that is coupled from the *n*-th eigenvectors in **U**.

### One ring (OR) model

The OR model denotes a communication scenario where the scatterers are located on a ring surrounded the user. The covariance matrix **R** in the one ring model is expressed as

(33) \begin{eqnarray*}\mathbf{R}= \frac{1}{2\omega } \int \nolimits \nolimits _{-\omega +\theta }^{\omega +\theta }{e}^{-j2\pi D(m-n)\mathrm{sin}(\Lambda )}d\Lambda .\end{eqnarray*}

where Λ represents the intervals/ranges of the AoAs distribution, parameter *D* the antenna spacing, *ω* denotes the angular spread, and *θ* represents the angle of arrival. It is worth noting that the integration in Eq. (33) is determined numerically.

### Laplacian (Lap) model

This subsection considers a local scattering model with Laplace distributed deviations. In particular, the covariance matrix **R** in the Laplacian (Lap) model can be obtained as

(34) \begin{eqnarray*}\mathbf{R}= \frac{1}{\sqrt{2}\omega } \int \nolimits \nolimits _{\theta -\pi }^{\theta +\pi }{e}^{- \frac{\sqrt{2}}{\omega } (\Lambda -\theta )}{e}^{-j2\pi D(m-n)\mathrm{sin}(\Lambda )}\mathrm{d}\Lambda .\end{eqnarray*}

For both the OR and Lap models, when the elements of BS are closely spaced and with limited scattering around the user, some of the eigenvalues in **R** become close to zero. It’s worth noticing that the **R** has a low-rank structure due to the limited angular spread. This allows a strong spatial correlation between the various pathways that manage the BS’s and users’ communication environment. The full-rank covariance matrix is produced using the exponential channel correlation model, which will be detailed in the following part.

### Exponential model

The exponential model discussed here provides a full rank **R**. As such, the (*m*, *n*)th element of **R** with an exponential model can be written as Loyka (2001) and Croisfelt Rodrigues, Marinello & Abrão (2019)

(35) \begin{eqnarray*}\mathbf{R}= \left\{ \begin{array}{@{}ll@{}} \displaystyle {r}^{n-m}, &\displaystyle m\leq n,\\ \displaystyle ({r}^{m-n})^{\ast }, &\displaystyle m\gt n, \end{array} \right. \end{eqnarray*}

where *r*(0 ≤ |*r*| ≤ 1) represents the correlation coefficient. The eigenvalue dispersion of the channel covariance matrix is represented by the correlation factor *r*. As such, in the exponential correlation model, raising the factor *r* leads to stronger spatial correlations.

**Figure 3:**
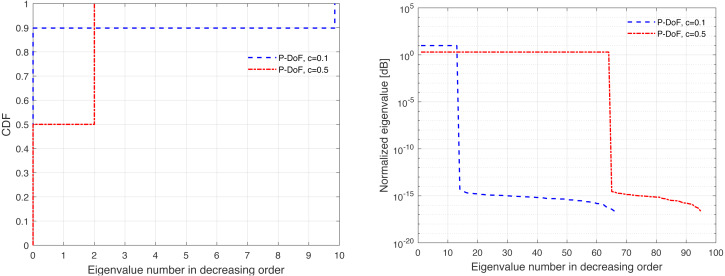
Illustration of the correlation in the *P*-DoF model.

**Figure 4:**
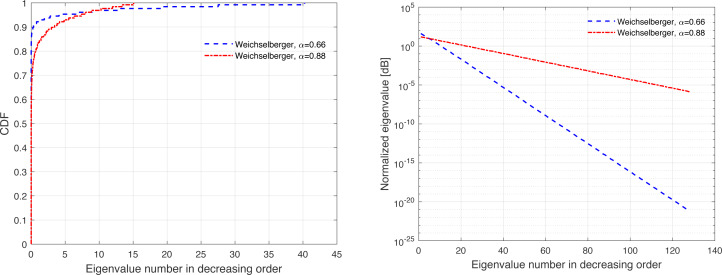
Illustration of the correlation in the Weichselberger model.

**Figure 5:**
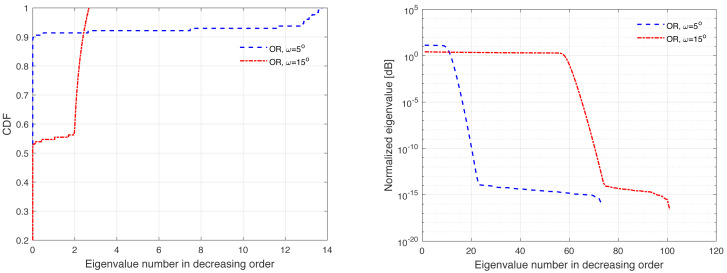
Illustration of the correlation in the OR model.

**Figure 6:**
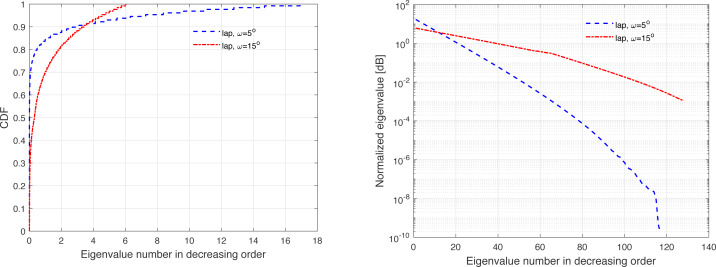
Illustration of the correlation in the Lap model.

**Figure 7:**
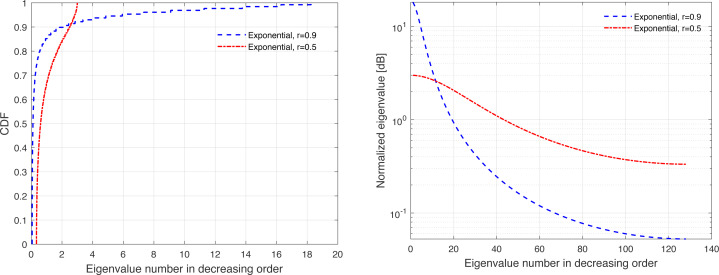
Illustration of the correlation in the Exponential model.

The channel covariance matrix’s eigenvalue distribution can be considered as a metric for quantifying the level of correlation. For instance, a very weak or no correlation denotes an identical eigenvalue distribution for all users, but large correlations suggest a small fraction of eigenvalues, which can be dominated and the remainder of the eigenvalues are almost zero. Furthermore, substantial eigenvalue changes are explained by strong correlations. Figures 3, 4, 5, 6, and 7 are provided to illustrate the effect of spatial channel correlation. These figures present the eigenvalues distribution in the *P*-DoF, Weichselberger, OR, Lap, and Exponential models, respectively. Different level of correlations are presented in each correlation model. The results show that even with a very strong correlation, Exponential model provide a full-rank covariance matrix since the normalized eigenvalues are not closed to zero. In what follows, we investigate the effect of channel hardening on the massive-MIMO systems.

## Channel hardening in massive-MIMO systems

Small-scale channel fading is one of the most common flaws in wireless communication systems. Random fluctuations in the channels are generated by microscopic changes in the propagation conditions. The oscillations render the channel gain unstable, because the channel gain might be very small, causing the broadcast data to be received incorrectly. To prevent small-scale fading, signal diversity can be performed. In particular, the diversity is achieved by delivering a signal over many channels with independent realizations. Besides, the spatial diversity can be simply achieved by deploying many antennas at the BS and/or at the receivers. Spatial diversity leads to channel hardening in massive-MIMO systems with a massive number of BS antennas. In simple terms, channel hardening means that a fading channel behaves as if it were not fading, implying that the channel gain after beamforming is nearly constant (Hochwald, Marzetta & Tarokh, 2004). As such, we may have a highly deterministic channel gain because the channel hardening could increase the reliability of communication networks. A propagation channel provides **h**_*k*_ asymptotic channel hardening if

(36) \begin{eqnarray*} \frac{{|}{|}{\mathbf{h}}_{k}{|}{{|}}^{2}}{\mathbb{E}\{ {|}{|}{\mathbf{h}}_{k}{|}{{|}}^{2}\} } \rightarrow [N\rightarrow \infty ]1\end{eqnarray*}

The expression in Eq. (36) implies that the channel gain approaches its mean value when the number of antennas at the BS grows very large. In this article, we consider the variance to illustrates how the channel and its means is closed to channel hardening so that

(37) \begin{eqnarray*}\mathbb{V } \left\{ \right. \frac{{|}{|}{\mathbf{h}}_{k}{|}{{|}}^{2}}{\mathbb{E}\{ {|}{|}{\mathbf{h}}_{k}{|}{{|}}^{2}\} } \left( \right. = \frac{\mathbb{V }\{ {|}{|}{\mathbf{h}}_{k}{|}{{|}}^{2}\} }{(\mathbb{E}\{ {|}{|}{\mathbf{h}}_{k}{|}{{|}}^{2}\} )^{2}} = \frac{ \left( \right. \text{tr}(\mathbf{R})^{2} \left( \right. }{ \left( \right. \text{tr}(\mathbf{R}){ \left( \right. }^{2}} ,\end{eqnarray*}

where the variance expression in Eq. (37) should be close to zero as *N* goes to infinity. The expression in Eq. (37) implies that the correlation, denoted by the eigenvalue variation of **R**, increases the variance and thus reduces the level of channel hardening.

**Figure 8:**
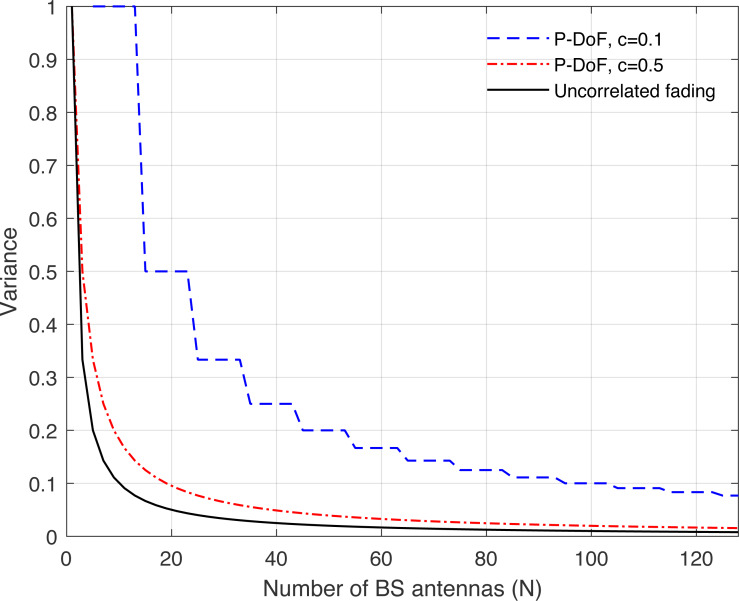
Illustration of the variance of the channel hardening phenomenon as a function of the number of BS antennas *N*. Uncorrelated fading is compared with the *P*-DoF channel model and under different correlation coefficients.

**Figure 9:**
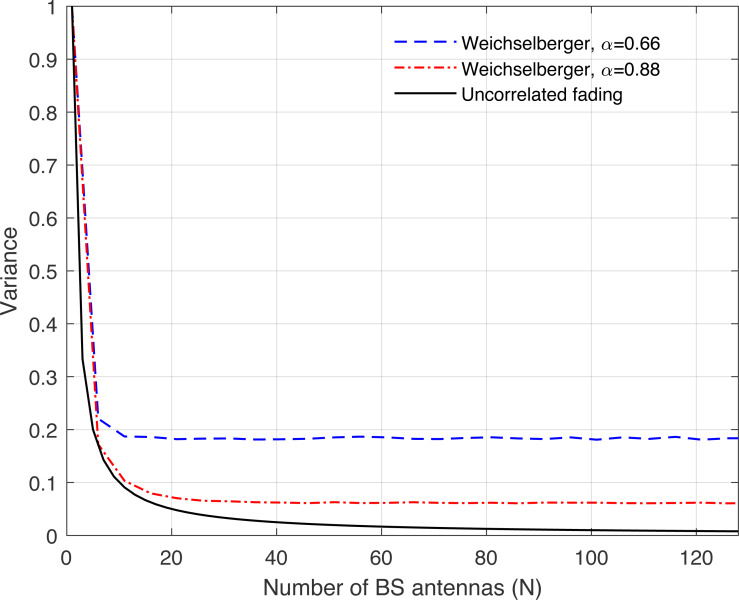
Illustration of the variance of the channel hardening phenomenon as a function of the number of BS antennas *N*. Uncorrelated fading is compared with the Weichselberger channel model and under different correlation coefficients.

**Figure 10:**
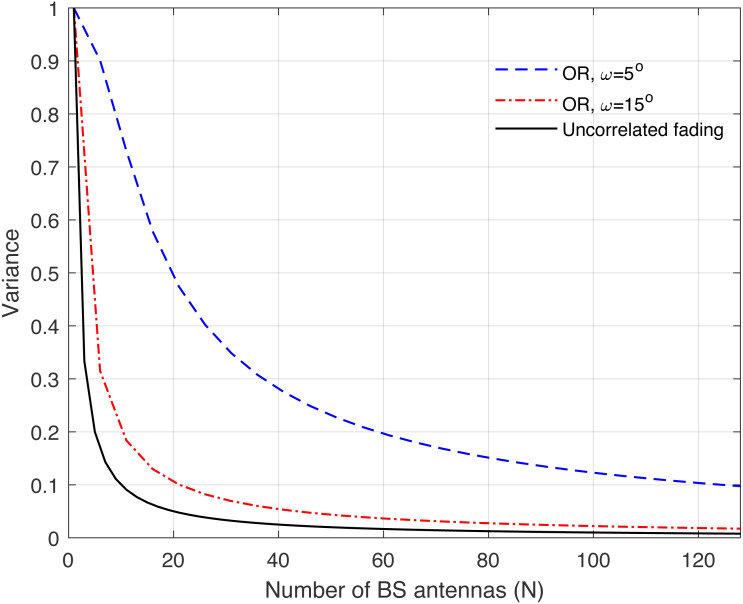
Illustration of the variance of the channel hardening phenomenon as a function of the number of BS antennas *N*. Uncorrelated fading is compared with the one ring (OR) channel model and under different correlation coefficients.

**Figure 11:**
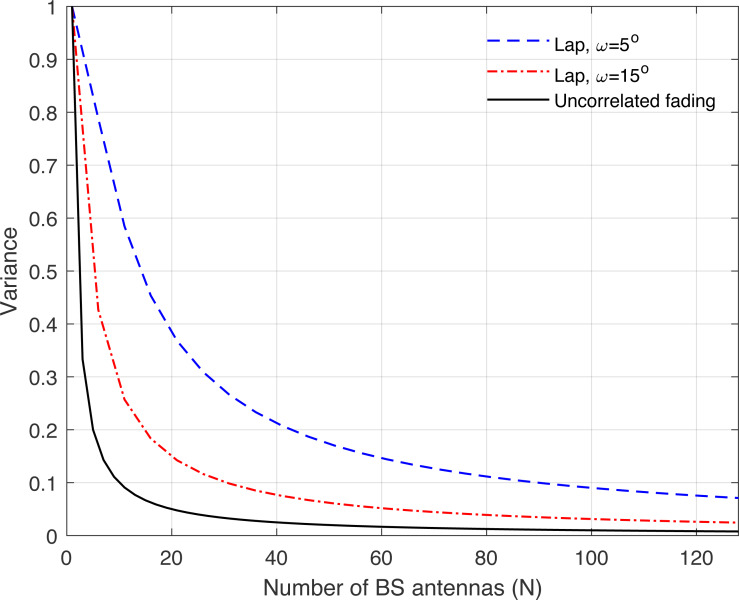
Illustration of the variance of the channel hardening phenomenon as a function of the number of BS antennas *N*. Uncorrelated fading is compared with the laplacian (Lap) channel model and under different correlation coefficients.

**Figure 12:**
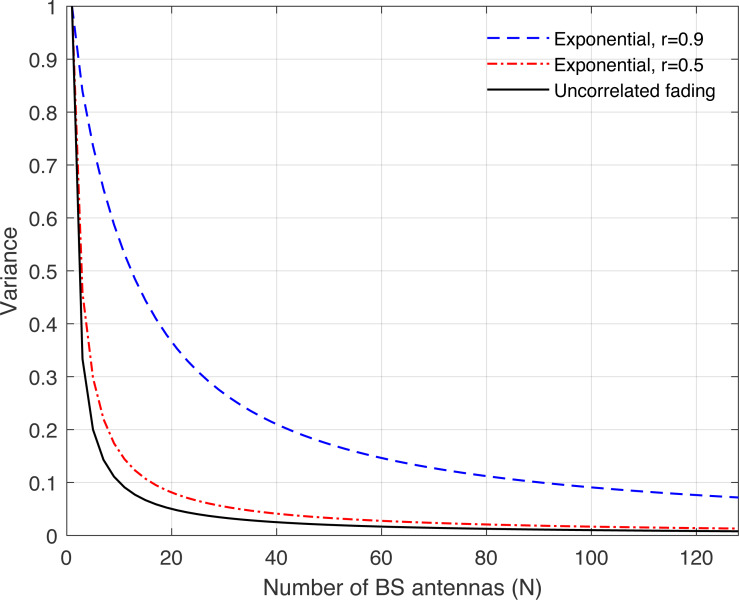
Illustration of the variance of the channel hardening phenomenon as a function of the number of BS antennas *N*. Uncorrelated fading is compared with the exponential channel model and under different correlation coefficients.

Figures 8, 9, 10, 11, and 12 are provided to illustrate the effect of channel hardening in the presence of correlation with different correlation models. The variance is obtained based on the expression in Eq. (37) as a function of number of BS antennas. The uncorrelated Rayleigh fading channel is compared with different correlation models. As can be observed, the smaller the variance, the more the channel is hardened. As expected, the lowest variance is achieved with uncorrelated Rayleigh fading channel. The results show that high correlation leads to a large loss in the channel hardening in all the correlation models considered.

## Numerical results and performance evaluation

This section presents some of numerical results, which examine the performance of massive-MIMO using FDD protocol. Specifically, the system performance is evaluated in terms of the ASR considering ZF precoding and the normalized MSE. In this present article, the results are provided for the uniform linear array configuration in different correlation models. Table 1 summaries the simulation parameters, which are considered in the performance evaluation. The MSE and ASR performances are averaged over 10000 independent channel realizations using a Monte-Carlo simulation. The number of training sequence length is selected based on the effective eigenvalues that are dominated. These effective eigenvalues for different correlation models are demonstrated in Figs. 3, 4, 5, 6, and 7, respectively. A short CT is considered with *τ* = 100 symbols. In this article, the training power *ρ*_p_ is assumed to be equal to the data power. However, optimization between the training and data powers are left to the future work, as stated earlier.

**Figure 13:**
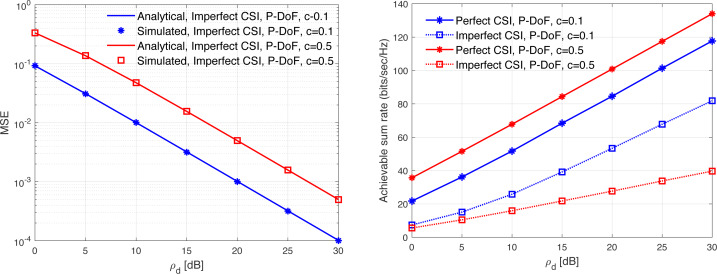
MSE and ASR as a function of the SNR *ρ*_d_ in dB using the *P*-DoF model with different correlation coefficients.

**Figure 14:**
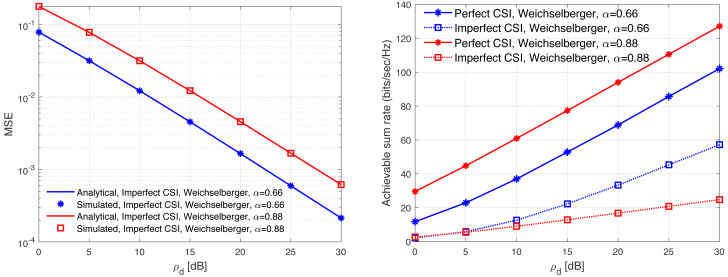
MSE and ASR as a function of the SNR *ρ*_d_ in dB using the Weichselberger model with different correlation coefficients.

**Figure 15:**
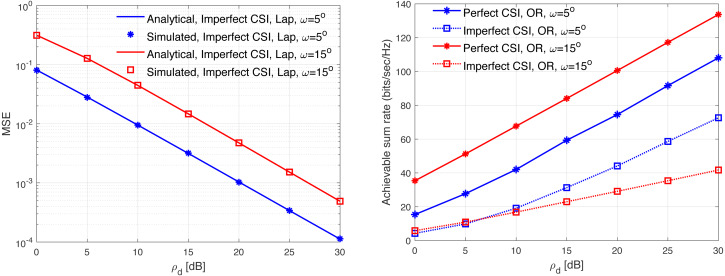
MSE and ASR as a function of the SNR *ρ*_d_ in dB using the OR model with different correlation coefficients.

**Figure 16:**
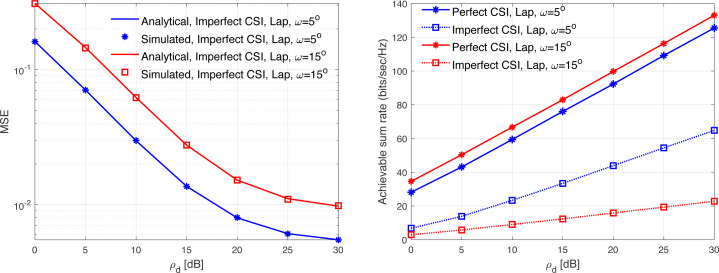
MSE and ASR as a function of the SNR *ρ*_d_ in dB using the Lap model with different correlation coefficients.

**Figure 17:**
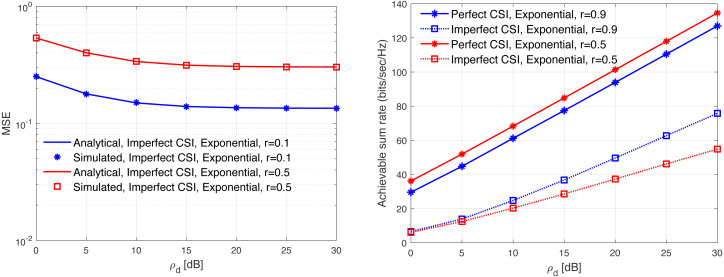
MSE and ASR as a function of the SNR *ρ*_d_ in dB using the exponential model with different correlation coefficients.

Figures 13, 14, 15, 16, and 17 demonstrate plots of the normalized MSE and DL ASR *versus* the SNR *ρ*_d_ in dB under different correlation models. The solid lines demonstrate the numerical analysis, while the markers represent simulation. The results demonstrate that an excellent matching between the simulated and the analytical results with various correlation models are obtained. The results show that the MSE of CSI estimation is reduced when the spatial correlation is increased. Besides, increasing the SNR improve the CSI estimation significantly. This is due to the fact that increasing the transmit power reduces the MSE performance. This is due to that fact that the error variance would approach zero in the high power regions. In addition, the error variance is reduced with the increasing of the spatial correlation. Specifically, when the level of correlation is relatively increased, the power in the channel is increased and the eigendirections denoted by the dominated eigenvalues is reduced. Consequently, this would provide a sufficient improvement in the CSI estimation accuracy even when a reduced training sequence length is used. Furthermore, the results indicate clearly the effect of carrying out DL CSI estimation. This is clearly observed in the plots when comparing the perfect CSI and imperfect CSI estimation. To this end, the results show the loss in the ASR is due the pre-log fraction, which effects the ASR considerably. The highest ASR with imperfect CSI estimation is obtained using the *P*-DoF and exponential channel models with *c* = *o*.1 and *r* = 0.9, respectively. Considering the perfect CSI estimation, the results clearly show that increasing the level of correlation reduces the ASR considerably under all the correlation models considered. In summary, the results indicate that minimizing the MSE of the DL CSI estimation does not maximizing the ASR in the limited CT. Using large arrays may enhance the spatial resolution and provide a sufficiently accurate CSI estimation. However, this is not necessary would enhance the ASR. Finally, the results provide an essential insight into the performances of the massive-MIMO systems with FDD transmission protocol and considering different spatial correlation models.

## Concluding remarks and future research directions

This article investigated the FDD massive-MIMO systems performance using different correlation models. To this end, statistical structure of massive-MIMO channel captured by the physical correlation is explored to find sufficiently accurate DL CSI estimation considering a short CT. The training sequence in DL is constructed based on the eigenvectors of the transmit correlation matrix. In this case, only the effective dominated eigenvectors is exploited to reduce the DL CSI estimation overhead. In addition, this article also examined the channel hardening phenomenon in the massive-MIMO systems considering FDD transmission protocol. The results showed that with strong correlation, a large loss in the channel hardening is obtained. Furthermore, the MSE minimization of CSI estimation and ASR maximization with short CT are evaluated under different physical correlation models. The results demonstrated that the spatial correlation minimizes the MSE of CSI estimation and can help in maximizing the ASR when short CT is considered. Future work may consider the investigation of the FDD massive-MIMO systems with high frequencies such as Millimeter waves and terahertz (THz) frequencies. Besides, exploiting the deep learning approach for CSI estimation could be possible in the future, see *e.g.*, Abdulwahhab & Jergees (2016), Ali & Taha (2021). Further, A resource allocation mechanism for future wireless communications networks (Fakhri et al., 2017) with massive MIMO systems could also be considered in future.

**Table 1:** Simulation parameters.

**Parameters**	**Symbol**	**Value**
Number of BS antennas	*N*	128
Number of users	*K*	10
Azimuth standard deviation	*ω*	5^∘^, 15^∘^
Antenna distance spacing	*D*	*λ*/2
CT	*τ*	100 symbols
AoA	*θ*	30^∘^

## Supplemental Information

10.7717/peerj-cs.1017/supp-1 Supplemental Information 1 Code used to generate the raw data

## Additional Information and Declarations

### Data Availability

The following information was supplied regarding data availability: The code and raw data are available as Supplemental Files.
